# Effect of Postconditioning With Intralipid in Patients Undergoing Off-Pump Coronary Artery Bypass Surgery: A Randomized Controlled Trial

**DOI:** 10.7759/cureus.80593

**Published:** 2025-03-14

**Authors:** Gegal Pruthi, Rajiv Kumar, Hemanthkumar Tamilchelvan, Naveen G Singh, Nagraj P. S., Anju Grewal, Praveen Choudhary

**Affiliations:** 1 Department of Anesthesiology, All India Institute of Medical Sciences, Bathinda, IND; 2 Department of Cardiothoracic Surgery, All India Institute of Medical Sciences, Bathinda, IND; 3 Department of Cardiac Anesthesiology, Sri Jayadeva Institute of Cardiovascular Sciences and Research, Bangalore, IND; 4 Department of Medicine, All India Institute of Medical Sciences, Bathinda, IND

**Keywords:** cardiac troponin-i, intralipid, myocardial protection, off pump coronary artery bypass surgery, postconditioning

## Abstract

Background: Off-pump coronary artery bypass (OPCAB) surgery is an alternative to conventional on-pump coronary artery bypass surgery for cardiac revascularization. It offers several advantages, including reduced mortality, decreased morbidity, and faster recovery. However, maintaining optimal myocardial function during OPCAB remains a challenge due to ischemia-reperfusion injury. Intralipid (IL), a fat emulsion used for parenteral nutrition, has shown potential cardioprotective effects. While the literature has elucidated IL's role as a preconditioning agent in OPCAB and a postconditioning agent in on-pump cardiac surgeries, its postconditioning role in OPCAB remains unexplored. This study aims to evaluate the postconditioning effects of IL in OPCAB surgery by measuring cardiac troponin I levels, hypothesizing that IL would reduce myocardial injury.

Methodology: Forty patients scheduled for elective OPCAB were randomly assigned to the IL group (L group) (*n* = 20) or the Control group (C group) (n=20) using a computer-generated randomization list. Group allocation was concealed in sequentially numbered opaque sealed envelopes. The IL group received a 20% IL infusion at 2 mL/kg (based on previous studies demonstrating cardioprotective effects at this dosage) immediately after revascularization, while the C group received an equivalent volume of normal saline. Cardiac troponin I (cTnI) levels were assessed preoperatively and at 6, 24, 48, and 72 hours postoperatively. Additionally, hemodynamic parameters during infusion, and various intraoperative and postoperative parameters (inotrope use, ventilatory hours, duration of intensive care unit stay, duration of hospital stay, postoperative left ventricular ejection fraction, postoperative lipid profile, renal and liver function tests) were evaluated.

Results: The results revealed no significant difference in cTnI levels between the intralipid and control groups at any postoperative time point. Postoperative lipid profile, as well as renal and liver function, remained unaffected in all patients.

Conclusions: As evidenced by comparable postoperative cardiac enzyme levels between the intralipid and control groups, we did not find a postconditioning role of IL in OPCAB surgery, suggesting that IL may not provide cardioprotection in this setting. The authors propose a further investigation into alternative administration protocols (like varying doses and administration timings of IL) to elucidate its potential impact as a postconditioning agent in OPCAB.

## Introduction

Off‐pump coronary artery bypass (OPCAB) surgery has emerged as an alternative to conventional on‐pump coronary artery bypass (ONCAB) surgery for cardiac revascularization. OPCAB offers distinct advantages, including the absence of an aortic cross-clamp, non-cannulation of the aorta, avoidance of cardioplegia, and maintenance of pulsatile flow. These factors contribute to reduced mortality, decreased morbidity, minimized cardiac enzyme release, shorter ventilatory support, and enhanced early recovery [1-6]. Despite these benefits, maintaining optimal myocardial function throughout the perioperative period primarily due to the risk of short-term ischemia-reperfusion injury episodes during intermittent coronary manipulation remains a challenge for anesthesiologists.

Preserving myocardial function involves strategies to enhance the heart's resilience against ischemic insult and subsequent reperfusion injury. These strategies encompass nonpharmacological methods such as mechanical approaches (intracoronary shunts) along with pharmacological interventions like anesthetic preconditioning and postconditioning techniques [7]. While various options exist, there is currently no universally accepted modality for myocardial protection in OPCAB.

The pathophysiology underlying myocardial damage during OPCAB during intermittent sequential short-term regional ischemic episodes (ischemic-reperfusion injury) results from the opening of the mitochondrial permeability transition pore (mPTP) [8]. Targeting this cellular mechanism of mPTP opening is considered a modality for effective myocardial protection. Intralipid (IL), a fat emulsion primarily used for parenteral nutrition, has demonstrated efficacy in treating cardiotoxicity induced by local anesthetic overdose [9]. While the literature has elucidated IL's probable role as a preconditioning agent in OPCAB and a postconditioning agent in On-pump cardiac surgeries, the potential postconditioning cardioprotective effects of IL during OPCAB remains unexplored [10-12]. Cardiac troponin I (cTnI) is highly specific to cardiac muscle and serves as a sensitive and reliable biomarker for detecting myocardial injury. Monitoring cTnI levels is crucial for diagnosing myocardial ischemia and assessing the extent of cardiac damage. This study aims to explore the unexplored cardioprotective effects of IL as a postconditioning agent in OPCAB by evaluating the postoperative cTnI levels, hypothesizing that IL would reduce myocardial injury.

## Materials and methods

Following approval from the Institutional Ethical Committee (IEC/AIIMS/BTI/383) and registration with the Clinical Trials Registry-India (CTRI/2023/11/060068), as well as obtaining informed consent, patients aged 18-70 years with a left ventricular ejection fraction (LVEF) >40% scheduled for elective OPCAB were enrolled from November 2023 to March 2024. Exclusion criteria included combined valve and coronary surgery, on-pump CABG, redo cardiac surgery, hyperlipidemia (total cholesterol ≥ 200 mg/dL or low-density lipoprotein ≥ 150 mg/dL), significant hepatic or renal dysfunction, uncontrolled hypertension, and immunological disorders (e.g., malignancy or positive HIV serology within the last six months). Patients with allergies to IL ingredients (e.g., eggs or soybean) and those receiving preoperative nicorandil or sulfonylurea were also excluded.

Sample size calculation was based on a study by Pruthi et al. [12], using openEpi version 3.0 (Dean AG, Sullivan KM, SOE MM OpenEpi: Open Source Epidemiologic Statistics for Public Health, www.OpenEpi.com), with mean ± standard deviation (SD) of 48-hour troponin levels of 0.11 ± 0.06 in the intralipid group and 0.32 ± 0.31 in the control group. A confidence level of 95% and a power of 80% were used. The calculated sample size was 18 patients per group. After accounting for an attrition rate of approximately 10%, 40 patients were recruited for the study, with 20 patients in each group. Forty eligible patients scheduled for elective OPCAB, performed by a single surgeon, were randomly assigned to the IL group (L group) or Control group (C group) using a computer-generated randomization list. Group allocation was concealed using sequentially numbered, opaque, sealed envelopes. The participants, cardiothoracic surgeon, data collector, and data analyst were blinded to the group allocation.

The association of any comorbidity and preoperative beta-blocker use was noted. Baseline assessments were measured before surgery, including echocardiography, liver function tests, renal function tests, serum lipid profile, and cTnI levels. Intraoperatively, total opioid usage and Minimal Alveolar Concentration of the inhalational agent were noted among both groups. The L group received an infusion of 20% IL (Fresenius Kabi, Homburg, Germany) at a dose of 2 mL/kg immediately after the completion of revascularization over 30 minutes, while the C group received an equivalent volume of normal saline over 30 minutes duration. Hemodynamic parameters were continuously monitored (and noted at the baseline before the initiation of IL or NS, at 5, 15, 30, 60, 120, and 180 minutes after initiation of infusion of IL or NS). Serum levels of cTnI were measured before surgery and at 6, 24, 48, and 72 hours postoperatively. Additionally, intraoperative and postoperative inotropic use was assessed using the vasoactive-inotropic score (VIS), calculated as follows: VIS = dopamine dose (mcg/kg/min) + dobutamine dose (mcg/kg/min) + 100 × epinephrine dose (mcg/kg/min) + 10 × milrinone dose (mcg/kg/min) + 10,000 × vasopressin dose (U/kg/min) + 100 × norepinephrine dose (mcg/kg/min). Ventilatory hours, length of intensive care unit (ICU) stay, and length of hospital stay were also measured. Postoperatively LVEF, lipid profile, liver function tests, and renal function tests were monitored on postoperative day (POD) 1 and POD 3. 

The primary outcome of the study was to evaluate the cardioprotective role of IL by assessing troponin I postoperatively at 6, 24, 48, and 72 hours in patients undergoing OPCAB surgeries. The secondary outcomes were to determine the hemodynamic effect of IL on systemic circulation; estimate the clinical effect of inotropic use (intraoperatively and POD-1) and postoperative LVEF; assess the effect of IL on the serum levels of blood lipids (triglyceride and total cholesterol), serum creatinine, and total bilirubin; evaluate the effect of IL on ventilatory hours and the length of ICU and hospital stay postoperatively; and investigate the outcome of complications occurring during hospitalization, including arrhythmias, stroke, infection, respiratory failure, hepatic or renal failure, reoperation, and mortality.

Statistical analysis

Data were expressed as mean ± SD. Comparisons between the groups were performed using an Independent t-test for continuous variables that followed a normal distribution and a Mann-Whitney U test for two groups that did not follow a normal distribution. Chi-square or Fisher’s exact test was used, as appropriate, for categorical variable comparisons between groups. Results were considered statistically significant at *P*-value ≤ 0.05. Statistical analyses were performed using IBM SPSS Statistics version 29.0.2.0 (IBM Corp., Armonk, NY).

## Results

**Figure 1:**
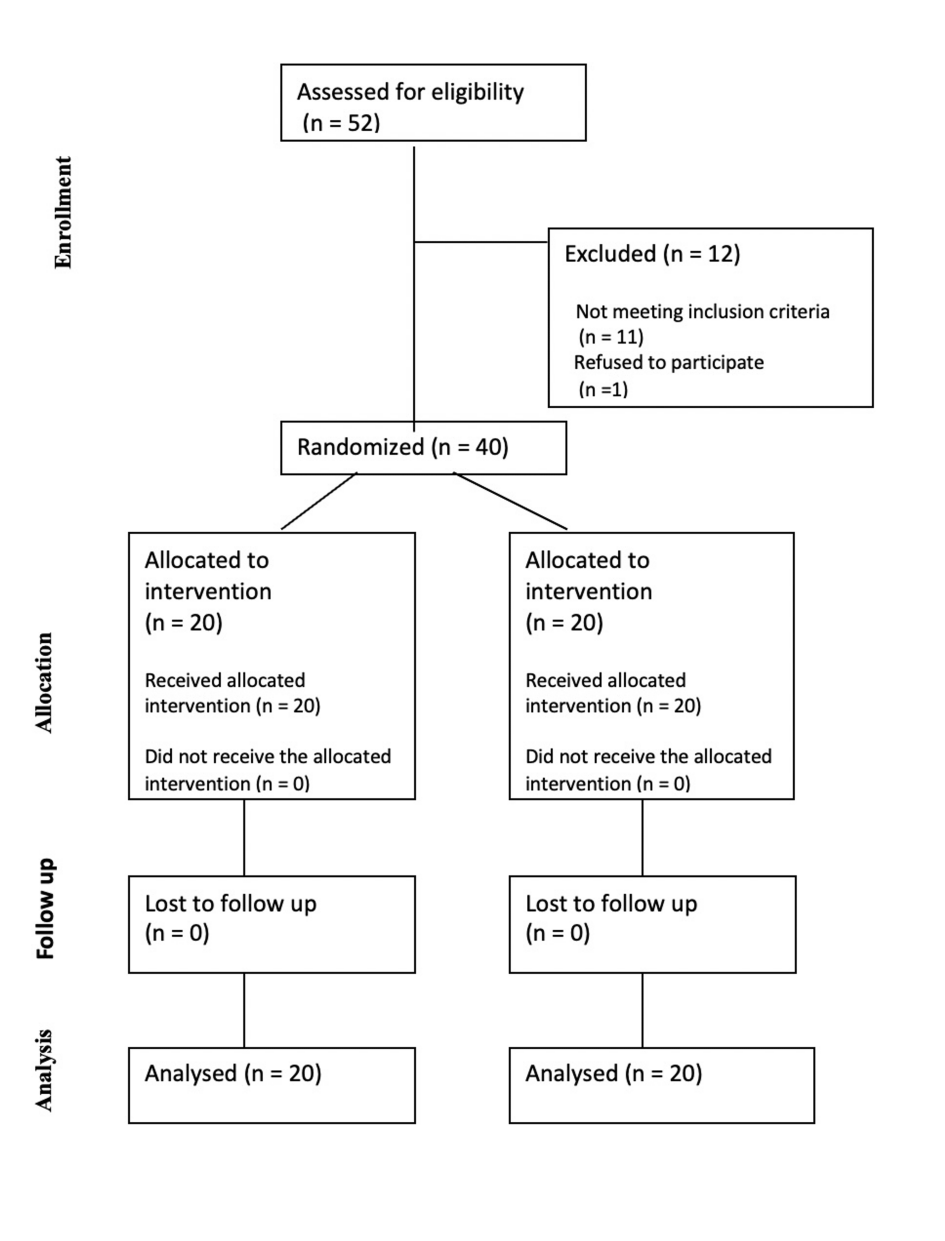
CONSORT flow diagram. CONSORT, Consolidated Standards of Reporting Trials

**Figure 2:**
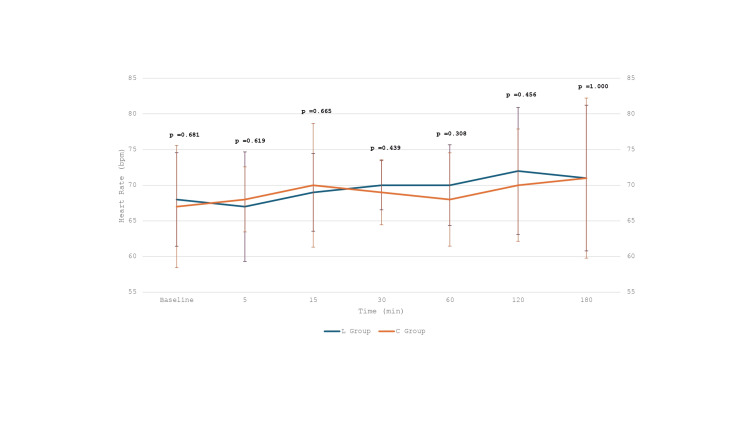
Intraoperative heart rate after initiation of infusion (Intralipid or normal saline).

**Figure 3:**
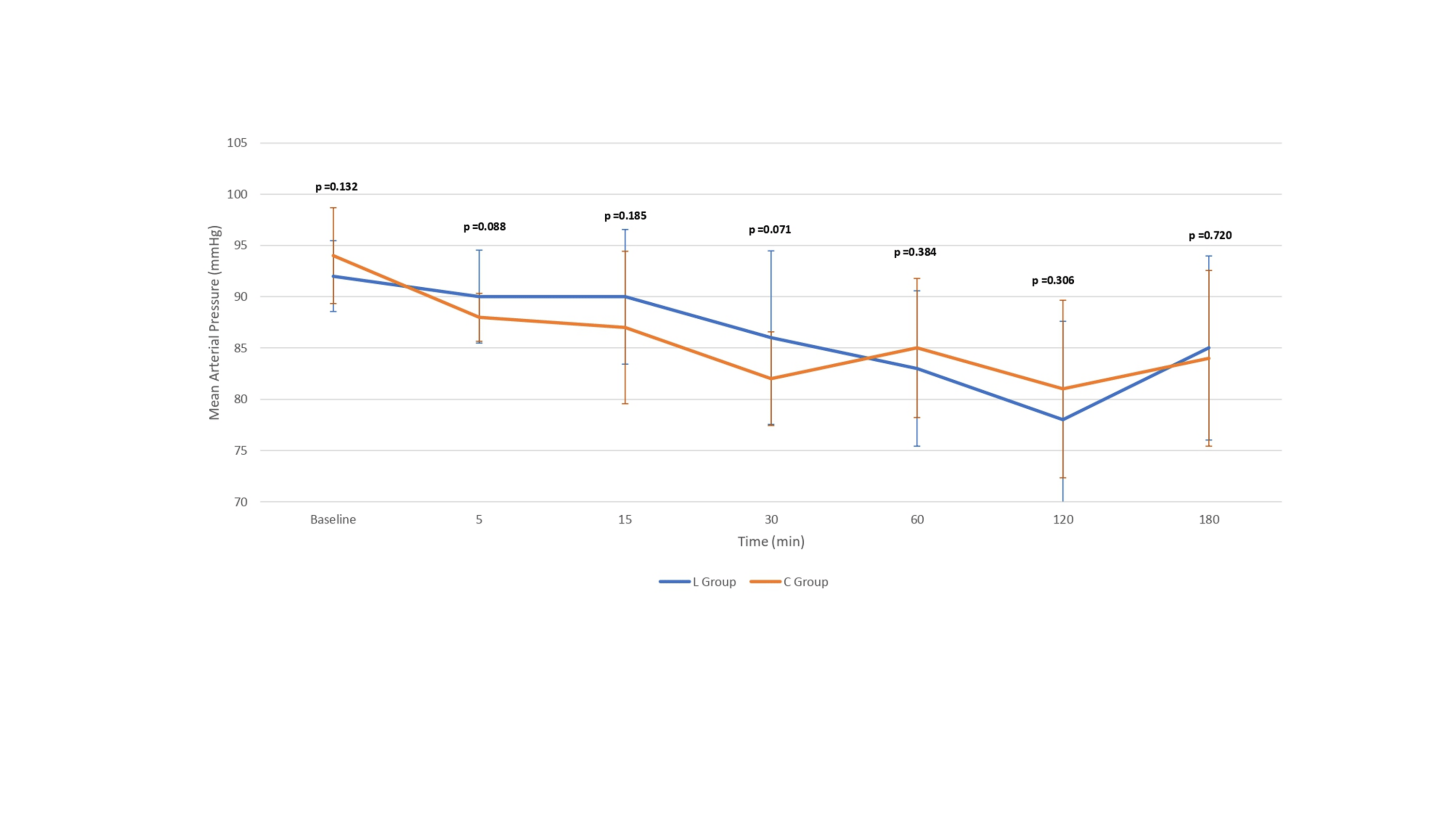
Intraoperative mean arterial pressure after initiation of infusion (Intralipid or normal saline).

Forty patients scheduled for elective OPCAB who met the inclusion criteria and provided written informed consent were randomly assigned to one of two groups (Figure 1). The study enrolled patients with comparable baseline characteristics in both the L group (*n* = 20) and the C group (*n* = 20) (Tables 1-2). Preoperative beta-blockers, aspirin, and statin use were comparable among both groups. Similarly, the use of inhalational agents and fentanyl during the perioperative period was comparable in both groups. There were no statistically significant differences between the L and C groups in baseline Troponin I levels or at any of the postoperative time points (6, 24, 48, and 72 hours), with *P*-values of 0.99, 0.34, 0.59, 0.79, and 0.97, respectively (Table 3). Hemodynamic parameters during IL or NS infusion (Figures 2, 3), intraoperative and postoperative VIS, postoperative LVEF, ventilatory hours, and length of hospital stay were also comparable among both groups (Tables 4, 5). There were no symptoms of acute myocardial ischemia, no new ischemic ECG changes, no development of pathological Q waves, and no new regional wall motion abnormality in any of the cases in both groups. None of the cases were converted to *on-pump*. However, an intra-aortic balloon pump (IABP) was inserted to support the procedure in three patients (two from the L group and one from the C group) with tight proximal LMCA stenosis. Biochemical parameters like lipid profile, liver function tests, and renal function tests on POD 1 were also comparable (Table 5). No complications were observed. Intraoperative opioid consumption and inhalational agent usage were similar between the groups (Table 4).

**Table 1:** Patient characteristics. M, male; F, female; HTN, hypertension; DM, diabetes mellitus; TVD, triple vessel disease; DVD, double vessel disease; SD, standard deviation

Patient characteristics	L group (*n* = 20)	C group (*n* = 20)	*P*-value
Age (years) (mean ± SD)	61.1 ± 8.08	58.45 ± 8.83	0.3284
Gender	M = 14	M = 12	0.5073
	F = 6	F = 8	
Height (cm) (Mean ± SD)	167 ± 8.86	163.3 ± 10.16	0.2272
Weight (kg) (Mean ± SD)	70.08 ± 16.26	68.57 ± 9.48	0.7217
Body mass index (kg/m^2^) (Mean ± SD)	24.49 ± 7.05	24.93 ± 6.55	0.8391
Preoperative beta-blocker use	Yes = 12	Yes = 13	0.7440
	No = 8	No = 7	
Comorbid conditions	HTN = 10	HTN = 12	0.5250
	DM = 7	DM = 10	0.3373
Coronary artery disease	TVD = 14	TVD = 13	0.7357
	DVD = 6	DVD = 7	
Number of grafts = 1	2	3	0.8854
Number of grafts = 2	6	6	
Number of grafts = 3	12	11	
Grafting time (Mean ± SD)	112 ± 17.89	109 ± 20.47	0.6245

**Table 2:** Preoperative patient data. SD, standard deviation

Patient characteristics	L group (*n* = 20)	C group (*n *= 20)	*P*-value
Baseline troponin I (ng/mL) (median, interquartile range)	0.01, 0.09	0.01, 0.09	0.9920 U = 199 (>127)
Baseline serum triglycerides (mg/dL) (Mean ± SD)	175.25 ± 97.37	137.03 ± 65.92	0.1543
Baseline serum cholesterol (mg/dL) (Mean ± SD)	138.95 ± 36.16	122.15 ± 61.40	0.2984
Baseline serum creatinine (mg/dL) (Mean ± SD)	0.921 ± 0.32	0.959 ± 0.33	0.714
Baseline serum bilirubin (mg/dL) (Mean ± SD)	0.629 ± 0.49	0.745 ± 0.47	0.450
Preoperative left ventricle ejection fraction (%) (Mean ± SD)	52.3± 7.05	48.6 ± 7.94	0.127

**Table 3:** Troponin I values postoperatively. SD, standard deviation

Troponin I (ng/mL)	L group (*n* = 20)	C group (*n* = 20)	*P*-value
Troponin I at 6 hours (ng/mL) (Mean ± SD)	0.332 ± 0.445	0.225 ± 0.215	0.3390
Troponin I at 24 hours (ng/mL) (Mean ± SD)	0.611 ± 1.028	0.452 ± 0.803	0.5889
Troponin I at 48 hours (ng/mL) (Mean ± SD)	0.664 ± 1.202	0.391 ± 0.649	0.7995
Troponin I at 72 hours (ng/mL) (Median, interquartile range)	0.016, 0.275	0.033, 0.335	0.9761

**Table 4:** Intraoperative patient data.

Intraoperative parameters	L group (*n *= 20)	C group (*n *= 20)	*P*-value
Fentanyl used (mcg) (Mean ± SD)	507 ± 41.18	490 ± 22.00	0.1117
Volatile agent used (minimum alveolar concentration) (median, interquartile range)	1, 0	1, 0	0.8415
Vasoactive inotrope score intraoperatively (Mean ± SD)	5.495 ± 4.095	5.19 ± 3.494	0.8013

**Table 5:** Postoperative patient data. POD, postoperative day; ICU, intensive care unit

Postoperative parameters	L group (*n* = 20)	C group (*n* = 20)	*P*-value
Serum triglycerides (mg/dL) (POD-1) (mean ± SD)	113.75 ± 49.89	110.1 ± 53.502	0.829
Serum cholesterol (mg/dL) (POD-1) (mean ± SD)	96.35 ± 25.59	109.4 ± 49.16	0.299
Serum creatinine (mg/dL) (POD-1) (mean ± SD)	0.974 ± 0.312	1.065 ± 0.506	0.498
Serum total bilirubin (mg/dL) xPOD-1 (Mean ± SD)	1.037 ± 0.511	1.162 ± 0.655	0.505
Vasoactive inotrope score (POD-1) (Mean ± SD)	4.12 ± 3.305	3.91 ± 2.282	0.816
Left ventricle ejection fraction (%) at the time of discharge (Mean ± SD)	52.7 ± 6.830	48.9 ± 7.80	0.109
Ventilatory hours (Mean ± SD)	8.07 ± 1.507	8.0 ± 1.622	0.888
Duration of ICU stay in days (Mean ± SD)	2.97 ± 0.952	2.75 ± 0.881	0.452
Duration of hospital stay in days (Mean ± SD)	5.4 ± 1.957	5.65 ± 1.725	0.671

## Discussion

Despite promising cardioprotective effects observed in other surgical contexts, the role of IL as a postconditioning agent in OPCAB surgery remains underexplored. This study investigates the potential cardioprotective effects of IL as a postconditioning agent during OPCAB surgery.

The period of ischemia during sequential coronary vascular occlusion in OPCAB surgery poses a risk of ventricular dysfunction, endothelial injury, and apoptosis, contributing to post-revascularization injury. This post-revascularization injury can adversely impact patient outcomes, highlighting the need for effective cardioprotective strategies. Ischemia-reperfusion injury triggers mechanisms such as calcium overload, oxidative stress, and adenine nucleotide depletion, ultimately leading to the opening of the mitochondrial permeability transition pore (mPTP) [8]. Inhibition of the mPTP opening has been considered a potential modality for myocardial protection. IL, a fat emulsion, has been known for its cardioprotective effects, but the precise molecular mechanisms are not fully elucidated. One proposed mechanism involves inhibiting mPTP opening through glycogen synthase kinase-3β via the PI3K/Akt/ERK pathway [11,12].

A study by Rahman et al. [11] highlighted the molecular mechanism of IL involving the inhibition of mPTP opening. In the study by Hu et al. [13], IL did not provide any noticeable myocardial protection when administered as a pre-conditioning agent. Derh et al. [14] delved further and evaluated the preconditioning effects of IL during off-pump coronary artery revascularization surgeries and they found decreased reperfusion injury in the myocardium as expressed by improvement in cardiac functions (LVEF and cardiac index) and normalization of cardiac markers (cTnI). This was in conjunction with another study done by Pruthi et al. [12] in which the role of IL as a preconditioning agent was evaluated and demonstrated as a safe pharmacological agent for OPCAB surgeries which can reduce post-ischemic myocardial injury (demonstrated by reduction in post-ischemic troponin T). Zhou et al. [15] reported the protective effects of IL against reperfusion injury when administered just before aortic cross-unclamping in valve replacement surgery.

Li et al. [16] found that the post-ischemic administration of IL is more effective in reducing the infarct size and improving cardiac functional recovery. Yu et al. [17] studied the effect of IL postconditioning on myocardial damage in patients undergoing valve replacement surgery with concomitant radiofrequency ablation for atrial fibrillation and observed that there was no beneficial effect of IL post-conditioning as the cardiac markers and AF recurrence was comparable between IL and C groups. Abdelhamid et al. [18] investigated the efficacy of IL as a postconditioning agent in patients undergoing on-pump coronary artery bypass graft surgery and found that post-ischemic reperfusion injury is reduced as evidenced by the reduction in the cardiac enzymes. 

The results of the present clinical trial did not reveal a significant postconditioning role of 20% IL (2 mL/kg) in OPCAB surgery, as evidenced by comparable postoperative cTnI levels between the L and C groups. The absence of a postconditioning effect, as evidenced by unchanged cTnI levels in the L group, could be attributed to the clinical course of certain patients within this group. Notably, two patients in the L group required IABP support preoperatively, compared to only one patient in the C group. The necessity of IABP in these patients might have contributed to the higher cTnI levels observed, reflecting more significant myocardial injury or stress. The other probable reason for this could be that there is no additional protective effect in administering IL, once mPTP pores are opened which occurs during or after revascularization as in OPCAB or on-pump surgeries. The complexities of ischemia-reperfusion injury during OPCAB surgery, coupled with the multifactorial nature of IL's mechanisms, necessitate ongoing exploration for refined and context-specific applications. The effect of an increased dose of IL could be explored further.

Future studies should explore varying doses, timings, and infusion durations to better assess IL's potential postconditioning effects. The present study employed a single bolus dose of 20% IL just after the completion of the anastomosis, within the range recommended for reversing local anesthetic cardiotoxicity [19]. It is worth noting that while the study provides valuable insights, limitations such as the modest sample size should be considered, and further research, exploring different doses and administration timings of IL for the postconditioning effect, is warranted to comprehensively assess its potential as a postconditioning agent in OPCAB.

## Conclusions

Although this study did not demonstrate a significant postconditioning role of 20% IL (2 mL/kg) in OPCAB as evidenced by comparable postoperative cTnI levels between the IL and C groups, these findings highlight the need to reevaluate its clinical utility and administration protocols in this setting. The lack of a postconditioning effect may be influenced by factors such as patient clinical course and the complexity of ischemia-reperfusion injury in OPCAB surgery. Despite these findings, further research with varying doses, timings, and larger sample sizes is needed to optimize IL's potential as a cardioprotective agent. These insights could guide future strategies to improve myocardial protection in OPCAB.
